# Mélanose de friction et habitudes vestimentaires

**DOI:** 10.11604/pamj.2018.30.215.16239

**Published:** 2018-07-17

**Authors:** Jawad El-Azhari, Mohammed Boui

**Affiliations:** 1 Service de Dermatologie-Vénérologie, Hôpital d’Instruction Mohammed V, Rabat, Maroc

**Keywords:** Mélanose de friction, habitudes vestimentaires, melhfa, hyperpigmentation, Friction melanosis, rubbing clothes, melhfa, hyperpigmentation

## Image en médecine

Friction melanosis is an acquired skin pigment disorder mainly affecting young subjects and occurring on areas next to bony prominences, in particular on the clavicular fossa and on the upper back. Repeated trauma due to friction of clothes on the skin over bony prominences are factors characterizing its pathogenesis. Other factors, such as darker skin phototype and individual variability may also play a role in pathogenesis. We report the case of a 40-year old female patient with skin phototype V and no notable medical history, presenting with non-pruritic brownish hyperpigmentation on the forehead, the cheeks, the chin and the neck evolving for several years. This woman from Morocco’s Sahara always weared her traditional clothing called “Melhfa“, consisting of an ample piece of fabric wrapped around the body to cover the hair, measuring approximately 4 meters. The examination of the remainder of the integument and of the mucous membranes as well as the remainder of the clinical examination and the laboratory tests were normal. The diagnoses of lichen planus pigmentosus, photodermatosis, macular amyloidosis or friction melanosis were suspected. Skin biopsy was performed, which objectified hyperpigmentation of the basal layer without amyloid deposits in the upper dermis. Biopsy was negative for Congo red stain. The diagnosis of friction melanosis was retained. The patient underwent topical hydroquinone 2% and avoided wearing Melhfa frequently.

La mélanose de friction est un trouble pigmentaire acquis touchant principalement les sujets jeunes sur les zones situées en regard des saillies osseuses, en particulier la zone claviculaire et le haut du dos. Les traumatismes répétés de frottement des vêtements contre la peau recouvrant les protubérances osseuses seraient fondamentaux dans sa pathogénie. D'autres facteurs, tels que le phototype foncé et la variabilité individuelle, peuvent également jouer un rôle dans la pathogenèse. Nous rapportons le cas d’une patiente de 40 ans sans antécédents notables, phototype V, qui consulte pour une hyperpigmentation brunâtre évoluant depuis plusieurs années, non prurigineuse et intéressant le front, les joues, le menton et le cou. Cette femme est originaire du Sahara marocain, et porte régulièrement une tenue traditionnelle appelée “melhfa“; tissu ample d’environ 4 mètres qui est enroulé autour du corps pour finalement couvrir les cheveux. L’examen du reste du tégument et des muqueuses ainsi que le reste de l’examen clinique et le bilan biologique sont normaux. Les diagnostics évoqués étaient un lichen plan pigmentogène, une photodermatose, une amylose maculeuse ou une mélanose de friction. Une biopsie cutanée a été effectuée objectivant une hyperpigmentation de la couche basale sans dépôts amyloïdes dans le derme supérieur, la coloration Rouge Congo était négative. Le diagnostic de mélanose de friction a été retenu, et la patiente a été mise sous hydroquinone crème topique à 2% en évitant le port fréquent de la melhfa.

**Figure 1:**
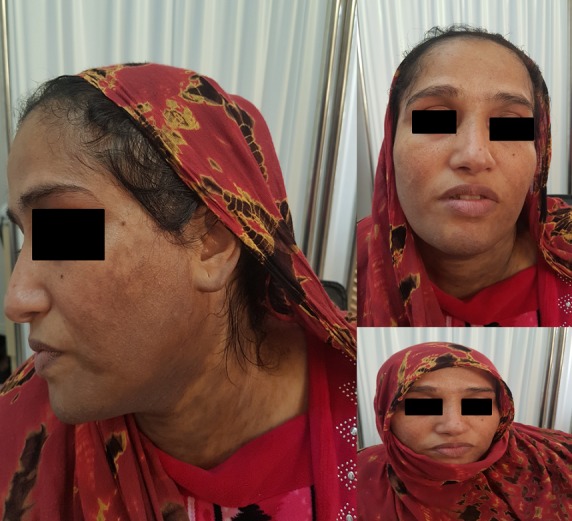
Hyperpigmentation brunâtre sur le front, les joues, le menton et le cou

